# Practical infection control training for Victoria’s aged care workforce at the time of COVID-19 pandemic: a community case study

**DOI:** 10.3389/fpubh.2023.1155980

**Published:** 2023-05-25

**Authors:** Samantha Dix, Helen Rawson, Philip Russo, Victoria Team, Debra Griffiths, Julia Morphet

**Affiliations:** School of Nursing and Midwifery, Monash University, Clayton, VIC, Australia

**Keywords:** COVID-19, education, health care professionals (HCP), infection control, personal protective equipment (PPE) compliance, program delivery, long-term care, workplace safety

## Abstract

The need to improve career development and training for residential aged care workers in Australia to achieve required essential competencies, including infection prevention and control competencies, has been repeatedly highlighted. In Australia long-term care settings for older adults are known as residential aged care facilities (RACFs). The COVID-19 pandemic has brought to light the lack of preparedness of the aged care sector to respond to emergencies, and the urgent need to improve the infection prevention and control training in residential aged care facilities. The government in the Australian State of Victoria allocated funds to support older Australians in RACFs, including funds toward infection prevention and control training of RACF staff. The School of Nursing and Midwifery at Monash University addressed some of these challenges in delivering an education program on effective infection prevention and control practices to the RACF workforce in Victoria, Australia. This was the largest state-funded program delivered to RACF workers to date in the State of Victoria. The aim of this paper is to provide a community case study, where we share our experience of program planning and implementation during early stages of the COVID-19 pandemic and lessons learned.

## Introduction

In Australia long-term care settings for older adults are known as residential aged care facilities (RACFs). Globally, studies indicate that even before the coronavirus (COVID-19) pandemic, RACFs were the most vulnerable institutions in terms of high incidence of infectious disease and suboptimal infection prevention and control (IPC) procedures (1). Since the beginning of the COVID-19 pandemic, researchers reported numerous coronavirus (SARS-CoV-2) infection outbreaks occurring in RACFs worldwide that affected both residents and staff (2, 3). SARS-CoV-2 can spread rapidly through RACFs if not managed appropriately (4, 5). The underlying factors for this transmission include: (1) the characteristics of the coronavirus pathogen (transmissibility, high replication and mutation rates), (2) the condition of hosts (residents’ older age, frailty and co-morbid conditions), and (3) transmission factors, including the ability to practice preventive behaviors (suboptimal IPC training of the RACF workforce, cognitive impairment of some RACF residents, personal protective equipment (PPE) availability, close-contact personal care) and built environment (close-contact living, shared communal areas and equipment) (4, 6–8). In addition, evidence from a rapid systematic review indicated that a larger facility size (number of beds), greater number of employees, staff availability, RACF staff operating between multiple facilities, and for-profit status of RACFs also contribute to the number and size of COVID-19 outbreaks in this setting (9). A systematic review of the causes of transmission and control measures of any pathogen outbreaks in RACFs indicated that the violation of basic IPC could play a major role in introducing and facilitating the spread of infectious diseases in RACFs (10).

IPC expertise in Australian RACFs is limited; and the COVID-19 pandemic highlighted significant gaps in IPC practices in facilities (7). A recent review reported that out of 134 RACFs, 44% of staff responsible for IPC had no specific IPC qualifications (11). Two independent reports into COVID-19 outbreaks in New South Wales and Victoria, Australia recommended improved continuing IPC training for staff in RACFs outside of outbreak situations, to be overseen by an appropriately trained member of the nursing staff (12, 13).

In Australia, The Royal Commission into Aged Care Quality and Safety (The Royal Commission) special report into COVID-19 in aged care (14) highlighted that the aged care workforce must be provided with regular IPC training, with the responsibility for this training resting with aged care providers. The Royal Commission called on the federal government to establish a national aged care plan for COVID-19 and deployment of infection control experts into RACFs as a condition of accreditation (15). In December 2020, the Australian Commonwealth Department of Health instructed all RACFs to appoint a nurse with appropriate accredited IPC training to lead IPC in their facility (16).

Staff training is important for effective IPC practices in RACFs (17). Infection prevention and control guidelines and training programs are often based on evidence collected in acute healthcare settings and not always relevant for the RACF context (18). Although IPC is the most commonly reported specialist skill among direct care workers in RACFs (19), little is known about the quality, relevance and frequency of training, or the undertaking of competency assessments (20). There are challenges implementing education programs in RACFs due to the diverse workforce with varied knowledge and educational experience, time to participate in education, and relevance, accessibility and sustainability of education programs (19, 21). Factors that may increase the efficacy of staff education in RACFs include a high-quality program using an interactive experiential learning format, that is relevant for staff and includes positive reinforcement and promoting sustainability (22).

The COVID-19 pandemic highlighted the lack of preparedness of the aged care sector in Australia to respond to emergencies and the urgent need to improve IPC training in RACFs among other challenges (23). The Victorian Government allocated funds to support people living in RACFs, including support for training the workforce in IPC practices (24). The School of Nursing and Midwifery at Monash University addressed some of these challenges through design and delivery of an education program on effective IPC practices to the RACF workforce in Victoria, Australia. This program was co-designed with nurses and direct care workers specifically for the RACF workforce and implemented an innovative education strategy, and evaluated its effectiveness to optimize IPC practice and protect people from healthcare-associated infections in RACFs. To date, this was the largest state-funded program delivered to the residential aged care workforce in the State of Victoria. The aim of this paper is to provide a community case study, where we share our experience of the program planning and implementation from the early emergency stages of the COVID-19 pandemic in Australia and the lessons learned.

## Context

### Setting and population

Since the beginning of the COVID-19 outbreak in Australia, residents in RACFs were considered at a high risk of COVID-19, leading to illness and loss of life (4, 5, 7). During the ‘second wave’ of COVID-19 from July to September 2020, there were over 2000 COVID-19 cases occurred within RACFs in the state of Victoria, which lead to almost 700 deaths (25). Older people in general (26, 27), and particularly those with co-existing illnesses, are at increased risk of severe infection, serious complications and increased case-mortality rates if they contract COVID-19 (28–33).

Based on the 2020 National Aged Care Workforce Census (19), 70% of the aged care workforce are Personal Care Attendants (PCAs), 23% are nurses and 7% are allied health professionals. The proportion of PCAs from culturally and linguistically diverse backgrounds (CALD) comprised over 62% (19). The majority (70%) of the PCA workforce were both migrant and spoke a language other than English; and almost three quarters (71%) of PCAs hold a Certificate III or higher qualification in a direct care field (19). The broad and diverse aged care workforce also includes auxiliary workers who may not have substantial infection control training; and as the outbreak progressed, there were surge workforce staff who may have been new to aged care. The delivery of care in RACFs is 24 h, 7 days a week and many workers concurrently worked in two or more RACFs (34).

### Program funders

The Victorian Government is committed to providing infection control training for the aged care workforce to help them adapt to the risky and changing environment posed by COVID-19. *The Coronavirus (COVID-19) Plan for the Victorian Aged Care Sector for Victoria* developed by the Department of Health and Human Services Victoria (DHHS) (23) provides guidelines to assist RACFs to support their workers, residents, families and visitors to prepare for prevention and management of COVID-19 individual cases and facility outbreaks. This project was funded by the Victorian Government through the DHHS.

### Program owners

Monash University, the owner of the program, is Australia’s largest university; and the School of Nursing and Midwifery is ranked 5th in the Academic Ranking of World Universities by Shanghai Ranking in 2022^1^. Monash Nursing and Midwifery is one of the largest educators of nurses and midwives in Australia, and has delivered nursing and midwifery educational courses for over 30 years, and graduated over 13,000 students.

## Key programmatic elements

### Program goal and objectives

The overall aim of this practical education program was to improve RACF staff IPC knowledge and practice, specifically related to appropriate donning and doffing of PPE to prevent transmission of COVID-19.

### Program components

This program incorporates three components: (1) practical face-to-face education on IPC knowledge and practices, including the application of PPE; (2) a train-the trainer model to facilitate sustainability of the program via facility champions; (3) and a virtual reality simulation, designed specifically for the aged care sector and used to consolidate knowledge.

Due to the nature of the evolving coronavirus pandemic, the education program was iteratively reviewed and updated to ensure alignment with both Australian and Victorian State Government guidelines and advice.

#### Practical face-to-face education session

With a focus on practical application of PPE, the face-to-face component of the program addressed the following key concepts related to infection prevention: COVID-19 transmission routes, current COVID-19 pandemic concerns specific to the aged care sector, and the role of health workers in prevention of COVID-19 transmission. The use of standard infection prevention precautions, including hand hygiene, face masks and physical distancing, and their effectiveness in preventing COVID-19 transmission were also covered. Further to this, the use of transmission-based infection prevention precautions, including different levels of PPE required and situations when it is used were a focus. Finally, the correct sequencing for donning and doffing PPE to avoid contamination of self, residents, or the RACF environment were also included in this program component.

This practical education session was conducted using contemporary education practices, including guided group discussion, active learning activities, and role play simulation. Pre- and post-session knowledge quizzes and observation of donning and doffing PPE using a structured checklist to assess each participant’s PPE application were used as assessments for learning. The education session was underpinned by a detailed lesson plan, which was used by all educators to promote quality and consistency in program delivery.

#### Train-the trainer model – Facility Champions

Following the practical component of the education program, additional education was provided to key RACF staff, nominated as IPC Facility Champions. This train-the trainer model was adopted as an effective strategy to equip the appointed Facility Champions with the ability to educate others in their organization. The core advantage of a train-the-trainer model is its cost and time effectiveness when providing education to large numbers, and a greater acceptance of content delivered by internal trainers enabling the facility to have an up to date content expert to assist with day to day challenges.

The train-the-trainer education session focused on the organization of the training sessions in RACFs, education practices during the training session, and accessing follow up support. Facility Champions were also able to further clarify IPC knowledge and practice if required. All education resources used during the face-to-face session, such as lesson plan, PowerPoint presentation, and handout materia were provided to the Facility Champions. Facility Champions were asked to upload a list of RACF staff they conducted training with to Monash University at the end of each session.

The Monash University education program coordinator contacted Facility Champions following their face-to-face session to provide support with ongoing training for their RACF staff. Facility Champions were able to contact the University training team *via* email for ongoing support as required; and all requests were attended within two business days.

#### Virtual reality simulation

To consolidate knowledge and build on sustainability of the education program, an online competency-based virtual reality simulation (VRS) was developed. The Monash University team worked closely with a commercial company with expertise in immersive technology and together a custom-built program of practice simulations was created. The VRS leveraged an advanced conversation engine allowing learners to have conversations with characters using artificial intelligence (AI). Mimicking real scenarios that aged care workers would face during healthcare delivery, the VRS facilitated consolidation of learning and complemented the face-to-face education. Designed purposefully and specifically for the aged care workforce, the VRS was accessible through a Windows-based personal computer PC or MAC. Enabling unscripted conversations between participants and AI characters that speak, listen, interact and are designed to replicate aged care staff, the VRS aimed to further support competency development and continued learning. On entering the VRS, participants were introduced to a simulated RACF environment and presented with a series of realistic clinical scenarios along with three different AI characters, a registered nurse, a PCA and an auxiliary staff member. The scenarios posed cases that required participants to make decisions related to prevention of infection, e.g., which level of PPE is required and the sequence of their use. Each AI character required instruction in the selection of appropriate PPE (transmission-based or standard precautions), and in the safe donning and doffing of PPE.

The VRS provided RACF staff with unlimited opportunities to practice instructions in donning and doffing of PPE, in a safe, low-risk setting, and to receive real-time feedback on the accuracy of their instruction, confirming information when it was provided correctly, and correcting inaccuracies. The VRS continues to be a sustainable source of relevant information, requiring fewer human resources while ensuring the quality of training delivery and enabling a depth of understanding. Staff are able to access the platform at any time of the day or night, including weekends. The VRS has been purposefully designed to be engaging for people of all language backgrounds and literacy levels, with cases tailored to ensure they reflect the diversity of the RACFs workforce. As such, the platform provided an inclusive and sustainable risk management strategy.

This education paradigm was chosen by the project team because it is engaging for users, consolidates learning, and assesses decision-making. This paradigm also has the added advantage of creating the basis for potential transformation of ongoing professional development for the aged care workforce, including *via* potential rapid delivery of new modules during times of crisis.

## Program planning, design and evaluation strategies

### Needs assessment

The education program was developed in response to discussions with the Victorian Government to help support Victoria’s RACFs during the second wave of COVID-19. A targeted IPC program that specifically focused on application of PPE in the setting of an ever changing COVID-19 infection landscape. The DHHS had already identified the need for this education in RACFs.

### Pilot

Prior to implementation of the education program, a pilot of the face-to-face education session was held with staff at a RACF in Melbourne, Victoria. Eight RACF staff attended: a mix of the facility care manager (a registered nurse), registered nurses (RNs), enrolled nurses (ENs) and PCAs. The aim of the pilot was to ensure the content of the education session was appropriate, engaging and useful for RACF staff, as well ensuring timing and sequencing was appropriate for the setting and intended audience. Participants most highly valued the opportunity to practice donning and doffing PPE. They also highly valued the ability to discuss IPC and PPE, and the challenges faced in the aged care sector, raising potential and actual challenges in relation to the prevention of COVID-19, with discussion related to what they would do if they had a confirmed case. The feedback provided from the pilot session was very positive. The participants valued the opportunity to identify the difficulties and develop possible solutions if a positive case of COVID-19 was detected in their RACF. Following the pilot, the face-to-face education session was refined and reduced to 90 min, with an additional 30 min allocated to the train-the-trainer session.

### Program evaluation strategies

The program operated under an education research design to ensure program quality and demonstrate outcomes. Data were collected concurrently with program delivery to inform iterative changes required to program delivery. We used a concurrent triangulation mixed methods design (35), and employed the following methods of data collection: course engagement, knowledge acquisition and application and translation to practice (Table 1).

**Table 1:** Program evaluation strategies.

Program evaluation strategy	Description
Course engagement	We recorded the number of participants who registered for, and completed, the face to face training, including basic demographic data, such as employing RACF and role in the RACF. Additionally, data was collected via follow-up at each RACF, to identify the number of staff members who subsequently received training from the Facility Champions. These data reflected the scale of the project, and informed areas to be targeted when engagement from RACFs reduced.
Knowledge acquisition/application	Each participant in the face-to-face training completed a short knowledge quiz at the beginning of the training, and again upon completion. We will compare knowledge scores pre- and post-training. Each participant was observed donning and doffing PPE. A structured checklist was used to assess participant application of PPE using the correct sequence. This approach enabled real-time feedback to be provided to each participant in the face-to-face training. Data from the VRS platform was collected, and aggregated to measure knowledge translation. Data collected included: number of times the platform was accessed, length of time spent on the platform, accuracy of instruction provided by participants, questions asked by participants of the human character. This enabled iterative changes to be made to the program, when common knowledge deficits were identified.
Translation into practice and follow up of compliance following the face-to-face session	Data were collected from a range of stakeholders related to translation of IPC principles into practice as follows: Education participants from the RACFs of Victoria Demographic data Post-education evaluation survey Education Facility Champions Post-education evaluation survey Residential aged care facility managers Post-education evaluation survey Monash University education team Field notes

### Risk mitigation plan

The COVID-19 pandemic has impacted the conduct of education provision, research and evidence synthesis of pandemic-related research projects that were launched at high speed in large numbers (36). Risk management at a University and project level became a key aspect of the project team’s approach to quality assurance (37). Monash University has a specialized Risk and Compliance Unit which facilitates risk management programs across the University. The University actions its risk management programs through a number of guidelines, policies and procedures including, risk assessment guidelines for major ventures and projects, fiscal misconduct policy, legal compliance policy and the risk management policy and procedures. Monash University recognized the significant safety and reputation risks associated with the delivery of the program. Five key risks were identified: (1) COVID-19 infection transmission during training; (2) difficulty or delays with educator recruitment; (3) failure to engage and improve understanding of the diverse residential aged care workforce; (4) insufficient reach across the sector to prevent outbreaks; and (5) inconsistency in education provided by Facility Champions. A risk mitigation plan was developed for all risks identified (Table 2).

**Table 2:** Risk mitigation plan.

Key risks	Proposed mitigation strategies	Roles
COVID-19 infections transmission during program implementation New outbreaks Loss of key educators Loss of key RACF workforce Risk for RACF staff and residents Reputation risk	A risk assessment to be put in place for all activities All practice to be aligned to Government guidelines including OHS controls on space requirements Educators to have and provide evidence of Influenza and COVID-19 Vaccination Screening of educators and participants for COVID-19 symptoms prior to each education session and any with symptoms to be asked to get tested and stay home Educators to receive training with academic oversight Preventive program (no overlap with crisis response)	Monash University primarily responsible DHHS to supply PPE that meets specifications in their policies and guidelines
Difficulty or delays with recruitment Delays to program implementation New outbreaks	Swiftly recruit core team of qualified educators Leverage networks to recruit Flexible recruitment responsive to flexible workforce (e.g., fractional and/or regional appointments) Set up logistics working group with HR representation Provide attractive salaries	Monash University primarily responsible DHHS to assist with communication
Failure to engage and improve understanding of diverse RACF workforce Risk for RACF staff and residents Breach of values (Monash University is inclusive) Reputation risk	Design and deliver a face to face education package tailorable to the experience and education level of attendees Develop inclusive and engaging VR/AI platform to support RACF staff in knowledge upkeep and dissemination (platform responsive to language backgrounds and accents)	Monash University primarily responsible
Insufficient reach across the sector to prevent outbreaks Safety risk for RACFs and residents Reputation risk	Employ dedicated personnel to manage relationships and booking with RACFs Target RACFs all over Victoria Hold 300 sessions at minimum	Monash University primarily responsible
Inconsistency in education provided by Facility Champions Misinformation New outbreaks Reputation risk	VR platform provides unlimited reinforcement of understanding of Facility Champions so they provide quality translation across other RACF staff	Monash University primarily responsible

## Results

### Implementation of the face-to face and train-the-trainer components

To facilitate smooth implementation of the education program, the following framework guided the approach: (1) provide a rapid response; (2) be safe; (3) be preventive; (4) deliver in person; (5) be inclusive; (6) be flexible; (7) create resilient RACFs; and (8) provide successful and sustainable outcomes (Table 3).

**Table 3:** Program implementation framework.

Practical approach	Description
Provide a rapid response	We will launch immediately with targeted invited RACFs (in collaboration with the Department to identify priority areas) and subsequently roll out a coordinated process for RACFs to opt-in, to book up to two education sessions for their staff in the first instance. The program applies to staff working in RACFs and this would include agency working therein. We will start with 2 teams of trainers (up to 10 sessions per week) and scale up to as many as 30 trainers (15 flexible, fractional teams, 60 or more sessions a week) as soon as possible as required to be responsive to RACF availability and flexible to the sessional nature of the workforce.
Be safe	Safety is our first priority. The program will align to the Coronavirus (COVID-19) Residential Aged Care Facilities Plan for Victoria, the Australian Government Department Coronavirus (COVID-19) guidelines for infection prevention and control in residential care facilities and the Communicable Diseases Network Australia (CDNA) National Guidelines for the Prevention, Control and Public Health Management of COVID-19 Outbreaks in Residential Care Facilities in Australia version 3.0 and applicable government restrictions. e.g., deliver training in environments with 4m^2^ per person, physically distanced to ≥1.5 m wherever possible, screening of trainers and participants. All educators will be required to have had the current influenza vaccination, will be screened for symptoms prior to conducting all education sessions, and will work in dedicated teams to avoid cross-contamination. Participants will be screened for symptoms at the beginning of each education session and no one with symptoms will be involved in the session; we will educate groups from one RACF at a time to reduce risk of cross-contamination and only hold sessions *in-situ* where it is safe to do so and adjust maximum group size accordingly; we will consider risk management and outbreak plan of the RACF and our internal risk assessment in determining the location (with assessment of room suitability including size).
Be preventive	We will work with RACFs without current or suspected COVID-19 cases; we will not overlap with the outbreak teams providing first response and crisis training. This will be considered at the time of booking and between booking and delivery of the session.
Deliver in person	We will deliver education face-to-face in person where we can build trust, any question asked can be addressed, and the use of Personal Protective Equipment (PPE) can be demonstrated, practiced and corrected.
Be inclusive	Any RACF (with the exception of those with a positive case of COVID-19) will be able to register with us for education; staff from any and all roles in aged care delivery and support are welcome to attend non-exclusively including Registered and Enrolled Nurses, Personal Care Attendants, clinical staff, lifestyle coordinators, laundry and kitchen staff, agency staff. The program will be accessible for a range of education and language backgrounds and literacy levels, to support workers at every level across RACFs. Users of the VR platform will have the ability to engage in the virtual environment in their level of English language proficiency – and in a high English language proficiency environment – this allows users to gain the ability to communicate about the course content with high English language proficiency, as is required in a crisis.
Be flexible	The COVID-19 situation is rapidly changing, the program will be regularly reviewed for scope to respond to education demands; we will work with RACFs to book appropriate times and training locations (*in-situ* where possible within physical distancing and local risk assessments and if required at Monash University campuses, or community centres/hospital education spaces with safety being the first priority in these decisions).
Create resilient RACFs	We will ask RACFs to nominate up to two Facility Champions (per session) which we will work with closely during the education session and empower with education materials to enable them to upskill staff at their facility; we will provide up to 300 RACFs with a VR platform and license for up to 20 staff to demonstrate an accessible, tailorable and swift education solution to support an agile workforce.
Provide successful and sustainable outcomes	Provide successful and sustainable outcomes: the program will be delivered through education research. The program is evidence-based, and the team will utilize a continuous evaluation process, enabling iterative development of the program based on outcomes from each course. Data to inform program evaluation will be collected *via* knowledge quizzes at the beginning and end of the program, a structured checklist assessing proficiency of donning and doffing PPE, and assessment of infection control decision-making using an VR platform.

A team of nurse academics from Monash University, with expertise and leadership in IPC, aged care, education evaluation research, clinical training development, and delivery, operations and logistics, worked together to rapidly co-design and implement this large-scale education program designed specifically for RACF staff across Victoria. The importance and urgency of rapid training of RACF staff in IPC and the evidence-based PPE use became heightened with the second wave of COVID-19 pandemic in Australia (7, 17). Prior to this, all available education and training in PPE application for the residential aged care workforce were primarily available online, based on evidence from acute healthcare and not fully adapted to the RACF setting and had low uptake and completion rates by workers.

The program was offered to all Victorian RACFs via an opt-in model and was promoted by the DHHS via newsletters and direct correspondence. A dedicated Monash University website^2^ was launched, enabling RACFs to directly register for the education program.

A team of educators worked in pairs to co-deliver each education session to a maximum of 20 participants per session. Within 3 months of commencement 309 face-to-face education sessions and train the trainer sessions were provided across 226 individual RACFs, including 159 RACFs located in Melbourne metropolitan area and 67 in regional Victoria. In total, 377 face-to-face education sessions were provided across 277 individual RACFs, including 170 RACFs located in Melbourne Metropolitan area and 107 in Regional Victoria. As part of this program, 4,219 RACF staff, including 1,207 Facility Champions completed the education (Table 4).

**Table 4:** Program implementation data.

Face-to-face training sessions delivered from August 2020 to October 2021	
Sessions commissioned and provided (total number)	
Sessions commissioned by the Department of Health and Human Services	370
Sessions provided	367
Sessions commissioned by Infection Prevention Control advice and response (IPCAR)	15
Sessions provided	382
Number of residential aged care facilities visited – geographic location	
Residential aged care facilities in Melbourne Metropolitan area	170
Residential aged care facilities in Regional Victoria	107
Total number of residential aged care facilities	277
Number of residential aged care facilities and other agencies visited – facility type	
Residential facilities	224
Public residential facilities	46
Supported Residential Service facilities	4
National Aboriginal and Torres Strait Islander Aged Care Program facilities	1
Community Housing facilities	1
Mental Health Hospital facilities	1
Torrens Agency	1
Aged Care Quality and Safety Commission (ACQSC)	1
Number of residential aged care facilities and other agencies visited – care type	
Facilities with high care beds	258
Total number of high care beds	16,652
Facilities with low care beds	134
Total number of low care beds	888
Program participants	
Total number of the program participants	4,175
Total number of Facility Champions participated in the program	1,207
Virtual Reality component	
Total number of facilities provided with Virtual Reality licences	301
Number of facilities (out of 301) have had a staff member activate at least one license	100
Total number of Virtual Reality licences provided to these 301 facilities	4,644
Number of Virtual Reality licences have been activated (out of 4,644 licences provided)	231

To evaluate the education program, participants were asked to complete a feedback survey 1–5 weeks after completion of their education session. Questions asked related to the usefulness of the session, changes made to IPC practice following the session and general feedback. Overwhelmingly, the feedback was positive with RACF workers reporting more understanding of IPC practices and their application within the facility. A snapshot of the participants’ responses to the process evaluation survey is presented in Table 5. Participants’ questions and educators’ concerns were regularly assessed by the project team; and the session content modified according with the raised needs (Table 5). Outcomes and impact evaluation of this program be presented in a subsequent publication.

**Table 5:** A snapshot from the process evaluation survey and feedback from the participants and educators.

Evaluation questions from post-education surveys	Summary of the participants’ responses	Examples of quotes
1. What changes (if any) have you seen in your practice of infection prevention following the education?	Participants reported that staff were more stringent in correctly donning and doffing PPE; they understood the use of PPE better; and there were changes in availability of PPE, such as P2/N95 masks.	“Donning and doffing with more understanding.” “More concentrated effort to get it right.” “We purchased nitrile gloves and changed the type of N95 masks and gowns we had. There is also more emphasis on ‘fit check’ when using N95 masks.”
	Participants reported changes in understanding of infection prevention principles, which may assist them in the future application of PPE across different clinical situations.	“Better understanding of why and how we do infection control.” “Continue infection prevention by being more aware of use of PPE.”
	Participants reported higher confidence in their practice when using PPE, along with more awareness of correct practices.	“Being more aware of my actions in regards to PPE and how to correctly follow the sequence.” “More confidence in ability to do things right.” “Definitely more confident and not as daunted.”
2. What was the most useful part of the education?	Participants overwhelmingly reported that the practical nature of the face to face sessions, especially being able to physically practice donning and doffing PPE was by far the most useful part of the session.	“Actively donning and doffing.” “The hands-on approach to learner made the session more beneficial and tailored to my learning style.” “The practical exercise of donning/doffing.”
	Participants reported it was useful to understand different elements of infection prevention and use of PPE, such as better understanding of hand hygiene practices, zoning and donning and doffing sequences.	“Use of alcohol-based hand rub.” “The understanding of clean and dirty areas.” “Correct sequence of donning and doffing of PPE.”
3. What was the least useful aspect of the training?	Most participants reported that all aspects were considered “very useful,” “effective” and “important.”	None provided in the participants’ replies
4. Do you have any other feedback for us?	Many participants stated that this program was a good refresher course in infection prevention principles related to the use of PPE; and they felt it was essential to keep up to date. They recognized the need to keep updating themselves with changes related to PPE use. They also reported the sessions were interesting, engaging and interactive and educators knowledgeable and approachable. Some would have liked further information related to specific issues faced in the aged care sector in relation to ICP.	“Good refresher of the course was well worth the time.” “Brilliant - could do refresher course once every 2–3 months – practice makes all difference.” “It’s good to keep updated to help keep people aware.”
**Questions and concerns raised by participants during education sessions**	**Specific questions during the education session**	**Response to the feedback**
Relevance and correct use of PPE within the RACF	The different levels of PPE use in different situations. The use of face masks and eye protection within the aged care facility. The availability of different types/levels of face masks. Correct application of P2/N95 masks. The use of eye protection – types and situations when it was required Wearing additional PPE, such as hairnets and booties. RACF staff often reported this as common practice at their facility. At times they were unsure about why they use this and how to don and doff safely when it is not included in the current sequence posters.	The requested information was incorporated in the future sessions and followed by a discussion with the participants.
Zoning and cohorting	RACF staff reported ongoing confusion about and asked questions related to how to zone and/or cohort residents in the setting of an outbreak.	Discussion related to application of ICP principles and challenges in an aged care environment to reflect these concepts.
Waste disposal and linen cleaning	Monash educators were often asked about correct practices related to waste disposal and linen cleaning	Discussion related to waste disposal and linen cleaning incorporated in future sessions.
**Questions and concerns raised by educators during education sessions**	**Specific concerns**	**Response to the feedback**
Ongoing inconsistencies in PPE practices	Eye protection – Variability in the use of and type of eye protection used in RACFs, including face shields and/or goggles, was observed	Discussion with participants during education sessions aimed to address these observations. Follow up discussion by the education team with RACF managers were also held.
	Face masks – Variability in the use a mask, including some RACFs limiting staff to two surgical masks per day and some RACFs allowing the use of cloth masks. In addition to this, there was ongoing confusion related to the use of P2/ N95 masks including of the correct practice completing a ‘fit check’.	
	Hand hygiene – Continued variability in hand hygiene practice, including understanding situations when hand hygiene should occur.	

### Implementation of the VRS component

Monash University commenced a targeted and intensive roll out of the VRS component in November 2020 to all RACF workers that had attended the face-to-face education session. Access to the VRS was *via* an individual coded license which gave workers unlimited access to the VRS software program.

A longer than anticipated time for testing and updating the VRS to align with emerging IPC knowledge and practice related to COVID-19 precautions meant there were some delays with the roll out of the VR component to RACF’s. In an effort to overcome these delays, the education team worked closely with RACF IPC leads and management in the roll out phase to facilitate license provision and VRS access for individual RACF’s. A staggered approach to RACF access allowed for follow up phone and video calls and face-to-face VR support sessions with RACF IPC leads and management. To incentivize RACF workers to engage with the VRS a $500 gift voucher was offered to the top four facilities with the most VR license activations.

## Discussion and conclusion

### Lessons learned

There have been a few lessons learned from this program design and implementation, which are important to document, analyze and share to improve further educational projects delivered to RACF staff. In the field of health program implementation, the discussion of the lessons learned traditionally consists of reflection on the three key questions *what went right*, *what went wrong*, and *how it could be improved* (38).

Acknowledging the contextual factors, this program was designed to address new reality of IPC and the use of PPE which COVID-19 brought to RACFs. This reality was evolving and changing in line with the SARS-CoV-2 adaptation, the stage of the pandemic, and new rapidly-attained evidence on COVID-19 infection control and prevention (39). These contextual factors impacted project planning and implementation. The importance of rapid training of RACF staff in IPC and the accurate use of PPE became heightened with the second wave of COVID-19 pandemic in Australia (7, 17); and the project team had limited time for planning the project. Nevertheless, effective leadership and the project team’s previous experience in delivering healthcare related education, including state-level projects allowed for successful planning, including the design of the multi-component training program, the development of the risk-mitigation plan, and adoption of the practical implementation approaches. A KPMG report (40) on program management in COVID-19 reality emphasized the importance of clarity of the project scope and delivery structures and the role of the project leadership. Other key lessons emphasized in this report (40), and also observed by us during the implementation process, were the need for stakeholder engagement, effective use of resources and successful management of the project phases, ensuring flexibility in altering schedules to accommodate changing needs. Well established research-RACF community collaborations between Monash University and RACF management and stakeholders’ direct interest in improved IPC skills of RACF staff were the main factors that facilitated successful implementation of the Program. The direct responsibility of the RACF management for IPC training of their staff (14) enabled the project team to run training sessions during the most challenging time when many staff were either sick or quarantined and the need for direct care was prioritized over training as the remaining staff were overworked (17).

The program design, specifically intended for the RACF setting, and particularly the multi-component program structure, contributed to successful program implementation despite the fact that some components were not engaged with as much as others by the RACF management and staff. The face-to-face training component, and particularly physical practice donning and doffing PPE, were acknowledged by the participants as the most useful parts of the program. The train-the trainer component was adopted as an effective strategy to equip the RACF Facility Champions with the ability to educate others in their organization and ensure program sustainability. The core advantage of a train-the trainer model is its cost and time effectiveness when providing education to large numbers, a greater acceptance of content delivered by internal trainers; and certainty the facility has an up to date content expert to assist with day to day challenges (41–43). Despite effective implementation of face-to-face, and train-the-trainer components, the VRS component was not well accepted. As already discussed, the unanticipated challenges included the lower than expected level of computer literacy among participants and issues with access to computers. Although the project team decided to incentivize the use of the VR component, this approach did not work given that the barriers to its implementation were not financial.

VR is an effective teaching/learning strategy, which is well established and is increasingly used in health professions education to improve procedural skills, technical knowledge and proficiency, and psychomotor skills (44–48). Acceptability and perceived usefulness of VR programs may vary and depend on the ability of the VR program to meet the users’ needs and complexity of the VR platform. In Australia, VR-based education on empathy and understanding of the physical environment for dementia care workers reported that VR may differentially assist the participants of different age and English-speaking background (49).

### Program limitations

This education program was designed and implemented as an emergency response to the evolving impact of COVID 19 in RACFs rather than regular planned professional development. An education program that is purposefully planned for specific learners allows for development of deeper understanding and knowledge that can be applied in the workplace to improve patient care (50). The rapid nature of the development and implementation of this education program limited the impact of the use of the VRS as a sustainable education strategy, with implications for future program implementation.

During the roll-out of the VRS, a number of unanticipated challenges were encountered in engaging RACF staff; and current activation sits at 19% of RACFs. These challenges included lower than expected level of computer literacy among RACF workers and reported limited access to computers both during work time and outside of work. Compounding this, is the lack of dedicated professional development time for RACF workers, an issue highlighted in The Royal Commission (14). Large changes in staffing in RACFs during and following the COVID-19 pandemic has seen many RACF workers that completed the face-to-face education, no longer working in the sector. Finally, the introduction of a trained IPC lead nurse at all RACFs within the aged care sector [following the impact of COVID 19 in some RACFs and the COVID-19 Special report by The Royal Commission (14)], who have been focusing more on the requirements for their new roles and responsibilities including ensuring their IPC education qualifications are met and have not yet established program implementation/staff training in their RACF.

### Practical implications

The need to improve career development and training the RACF workforce in Australia to improve the required essential competencies has been repeatedly highlighted (51), including their IPC competencies (7, 17). Effective IPC training is essential for protection of residents and staff in RACFs not only during a pandemic, but also for routine care; however, it is often neglected (52). Improved IPC practices will help to reduce RACF financial costs related to the need to replace quarantined staff with agency staff, and employ additional staff to address the extra workload due to the increased acuity of care (52).

Education and training in the aged care sector are often based on evidence collected in acute healthcare settings and adapted for their use in RACFs, where the needs of patients and educational level of staff are significantly different to that of RACFs. These programs are not always relevant for the RACF context (18), making it difficult for staff to translate knowledge and understanding gained from the education to care of residents in RACFs. Therefore, well planned education programs specifically designed for RACF workers would be meaningful and beneficial for future education practice.

Adding to this, the RACF workforce development in Australia is a complex issue, as RACF staff do not have time away from care tasks to attend training and access educational resources (53). Previous studies also discussed the RACF staff diversity and highlighted the need for equitable access to educational resources for staff from non-English speaking backgrounds (53). The lack of clear pathways for RACF workers to develop their knowledge and skills and advance within the sector has also been acknowledged (54). In addition to attracting and retaining, RACF staff, education and training in Australia becomes an increasingly important area of concern (54).

This program was implemented in the beginning of COVID-19 outbreak when a State of Emergency was declared in Victoria. At that time, knowledge of the impact of the pathogen and its transmission routes were limited. It is important to note that, in addition to common worries about their own and their family’s health and life, RACF workers were anxious about transmitting COVID-19 infection to residents (14). This situation was the main driver of the program uptake by RACFs, potentially reducing motivation for ongoing education related to IPC practices after the State of Emergency was lifted.

We shared this community case study to demonstrate that educational sector-aged care sector partnership enhanced the collaborative capacity of our project for the design, development and implementation of an education program specifically for the IPC training of RACF workers. Careful project planning and program co-design, strong leadership, effective communication with the project stakeholders and their engagement in the project, as well as process evaluation and program adaptation to reflect the participants’ needs and address the educators’ concerns, were the critical success factors that facilitated smooth implementation. The program provided direct feedback and support to industry partners, and optimized potentially life-saving procedures during a traumatizing time for the sector.

In regards to the programmatic elements, we aimed to highlight the RACF workers’ and managers’ preference for the traditional face-to-face and the train-the trainer components of the IPC training rather than VR component. In emergency situations, such as the COVID-19 outbreak, we suggest that developers of educational projects intended to upskill RACF staff use these traditional educational methods. However, the use of technology, such as VR, for education purposes in RACFs warrants further exploration.

## Ethical issues

An online survey using a secure web-based platform was used to collect pre-and post-quiz knowledge data. Completion of the quiz was anonymous and contained no identifying features. Participants used a QR code at the face-to-face session to access and complete the pre-quiz and were emailed the link to the post-quiz 3–6 weeks following completion of face-to-face education. Participant email addresses were provided to the Monash University PPE Project Administrator upon registration in the program.

VRS data was collected when participants accessed and interacted with the virtual reality platform using an individual access code emailed to each participant with the link to the post-quiz survey. Prior to entering the VRS, participants were required to complete a privacy statement regarding the collection and use of data for this activity. Data collected were de-identified, aggregated and analyzed to evaluate the PPE education program outcomes.

All survey and VRS data were stored securely in LabArchives, and were accessible only to the research team.

## Data availability statement

The original contributions presented in the study are included in the article/supplementary material, further inquiries can be directed to the corresponding author.

## Ethics statement

The studies involving human participants were reviewed and approved by the Monash University Human Research Ethics Committee (Project ID: 26516). Written informed consent for participation was not required for this study in accordance with the national legislation and the institutional requirements.

## Author contributions

SD, JM, HR, PR, and DG secured the grant. SD and VT conducted the literature search and drafted the manuscript with support and guidance from JM, HR, PR, and DG. All the authors critically reviewed and contributed to the individual parts of the manuscript, approved the final version, and agreed to be accountable for the content of this work.

## Funding

This project was funded by the Victorian Government through the Department of Health and Human Services Victoria (HHSD/20/350937).

## Conflict of interest

The authors declare that the research was conducted in the absence of any commercial or financial relationships that could be construed as a potential conflict of interest.

## Publisher’s note

All claims expressed in this article are solely those of the authors and do not necessarily represent those of their affiliated organizations, or those of the publisher, the editors and the reviewers. Any product that may be evaluated in this article, or claim that may be made by its manufacturer, is not guaranteed or endorsed by the publisher.

## Footnotes

^1^ https://www.shanghairanking.com/rankings/gras/2021/RS0404

^2^ https://www.monash.edu/medicine/enterprise/ppe-project
